# Differential Diagnosis of Azoospermia in Men with Infertility

**DOI:** 10.3390/jcm10143144

**Published:** 2021-07-16

**Authors:** Danilo L. Andrade, Marina C. Viana, Sandro C. Esteves

**Affiliations:** 1 Department of Medical Physiopathology (Postgraduate Program), State University of Campinas (UNICAMP), Campinas 13083-887, SP, Brazil; danilo_landrade@hotmail.com; 2 Department of Surgery (Residency Program), Division of Urology, State University of Campinas (UNICAMP), Campinas 13083-887, SP, Brazil; marinacorreaviana@gmail.com; 3 ANDROFERT, Andrology & Human Reproduction Clinic, Campinas 13075-460, SP, Brazil; 4 Department of Surgery, Division of Urology, State University of Campinas (UNICAMP), Campinas 13083-887, SP, Brazil

**Keywords:** azoospermia, diagnosis, male infertility, nonobstructive azoospermia, spermatogenic failure, testis biopsy, sperm retrieval, genetic testing, endocrine evaluation, review

## Abstract

The differential diagnosis between obstructive and nonobstructive azoospermia is the first step in the clinical management of azoospermic patients with infertility. It includes a detailed medical history and physical examination, semen analysis, hormonal assessment, genetic tests, and imaging studies. A testicular biopsy is reserved for the cases of doubt, mainly in patients whose history, physical examination, and endocrine analysis are inconclusive. The latter should be combined with sperm extraction for possible sperm cryopreservation. We present a detailed analysis on how to make the azoospermia differential diagnosis and discuss three clinical cases where the differential diagnosis was challenging. A coordinated effort involving reproductive urologists/andrologists, geneticists, pathologists, and embryologists will offer the best diagnostic path for men with azoospermia.

## 1. Introduction

Azoospermia (a-, without + –zoo– » Greek zôion, animal + –spermia– » Greek sperma, sperm/seed) is defined by the absence of sperm in the ejaculate. Although the term does not imply an underlying etiology, azoospermia inevitably provokes infertility [1]. According to global estimates, 1 out of 100 men at reproductive age and up to 10% of men with infertility are azoospermic [2,3,4].

Azoospermia is broadly classified into obstructive and nonobstructive. This differentiation is clinically meaningful because it affects patient management and treatment outcomes [4]. Notably, nonobstructive azoospermia (NOA) relates to an intrinsic testicular defect caused by various conditions that ultimately affect sperm production profoundly.

The severe spermatogenic deficiency observed in NOA patients is often a consequence of primary testicular failure affecting mainly spermatogenic cells (spermatogenic failure (STF)) or related to a dysfunction of the hypothalamus-pituitary-gonadal axis (hypogonadotropic hypogonadism (HH)). From this point on, the acronyms STF and HH will distinguish these types of NOA, as appropriate [5]. The above-proposed terminology might be more intuitive for the clinician. It not only indicates the site of the problem (central or local) explicitly, but also makes it clear that the testicular disorder refers primarily to a spermatogenic defect, unlike the indistinct term ‘testicular failure’ that may relate to an isolated spermatogenic defect or such a defect combined with Leydig cell failure.

The differential diagnosis between STF and HH is also essential because the former is linked with severe and untreatable conditions, whereas the latter can be effectively treated with gonadotropin therapy [5,6]. By contrast, obstructive azoospermia (OA) originates from a mechanical block along the reproductive tract, namely, vas deferens, epididymis, or ejaculatory duct [7,8]. Unlike NOA, spermatogenesis is preserved, and both reconstructive procedures and sperm retrieval are typically highly successful in OA patients [7,8,9,10].

Nonobstructive azoospermia can be distinguished from OA using history, physical examination, semen analysis, hormonal assessment, and genetic testing in most patients [4,5,11]. However, in some instances, this distinction is not straightforward, and a testis biopsy is required. In this article, we first provide readers an overview of the azoospermia differential diagnosis. Secondly, we discuss the differential diagnosis in cases of doubt, including a workable clinical algorithm. Lastly, we present exemplary clinical cases to illustrate a difficult diagnosis and its outcomes. 

## 2. Azoospermia Differential Diagnosis: An Overview

The primary goals of the differential diagnosis are the identification of:

- Potentially correctable forms of azoospermia (e.g., by surgery or medication).
- Irreversible types of azoospermia suitable for sperm retrieval and intracytoplasmic sperm injection (ICSI), using own sperm.
- Types of azoospermia in which donor insemination or adoption are the only possibilities.
- Health-threatening illness associated with azoospermia requiring medical attention.
- Genetic causes of azoospermia that may affect the patient or offspring’s health, mainly if assisted reproductive technology is used.

It is critical to evaluate the azoospermic patient using a standardized workup to achieve these goals, as discussed in the next sections.

### 2.1. Medical History

A thorough medical history is pivotal to help determine the type of azoospermia. It must cover eight critical elements (Table 1), which are:

1. Infertility history
2. Sexual history
3. Childhood and development history
4. Personal medical history
5. Previous surgery/treatments
6. Gonadotoxic exposure
7. Family history
8. Current health status and lifestyle

The history may reveal the presence of congenital abnormalities, such as cryptorchidism, which could result in NOA-STF. Testicular infections (e.g., mumps orchitis), testicular trauma, testicular torsion, gonadotoxin exposure (e.g., radiotherapy/chemotherapy, anabolic steroid use, testosterone replacement therapy), or a history of brain surgery are informative to help establish a possible etiologic factor for NOA [5]. Hypogonadotropic hypogonadism is caused by congenital (e.g., Kallmann syndrome) or acquired conditions (e.g., prolactinomas, pituitary surgery, or testosterone replacement therapy) [6,12,13]. Notably, testosterone injections—commonly prescribed nowadays to men at reproductive age with signs of hypogonadism—suppress the hypothalamic-pituitary-gonadal axis. Consequently, intratesticular testosterone levels—critical for normal spermatogenesis—remain very low and, therefore, unable to sustain spermatogenesis [14].

On the other hand, a history of hernia repair, scrotal surgery, pelvic surgery, endoscopic urethral instrumentation, or genitourinary infection (e.g., epididymitis) may cause OA. Along these lines, a previous vasectomy is a typical OA etiology [8]. However, in many cases, the etiology cannot be determined, and additional tests are required, as explained in the following sections.

### 2.2. Physical Examination

The physical exam is critical in the assessment of men presenting with azoospermia. It starts with the appraisal of the overall body characteristics, with a focus on secondary sexual characteristics. Abnormal body hair distribution and gynecomastia may be indicative of hypogonadism or hormonal disturbances [5,11]. Examination of inguinal and genital areas may unveil scars from previous surgeries that could have injured testicular blood supply and the vas deferens. Other physical defects, such as abnormalities of the penis (e.g., hypospadias, epispadias, short frenulum, phimosis, fibrotic nodules), should also be evaluated.

Testicular size, texture, and consistency should be assessed. In routine practice, testicular volume is estimated using the Prader’s orchidometer. The mean testicular volume measured using the Prader’s orchidometer in the general population is 20.0 ± 5.0 mL [15]. 

Testes of men with OA have a firm texture. About 85% of testicular parenchyma is implicated in spermatogenesis. By contrast, men with NOA usually have small testicles (<15 mL or ≤4.6 cm long axis) [16]. However, it should be noted that there is no threshold for testicular size to completely exclude the possibility of harvesting sperm on a retrieval attempt [17]. Moreover, both patients with OA and NOA-STF due to maturation arrest have normal-sized testicles [18]. Therefore, testicular size may not necessarily be informative for the differential diagnosis. Palpable abnormalities of the testis should be further evaluated with imaging studies because azoospermic men, particularly those with NOA-STF, have increased risks of developing testis malignancy [19].

The presence of the vas deferens and the epididymis’ characteristics should always be determined. A normal and healthy epididymis is firm, whereas an obstructed epididymis is ingurgitated (soft) [11]. Patients with NOA typically have palpable vasa deferentia and flat epididymides [5].

The vas deferens is easily palpable inside the spermatic cord as a firm, round, “spaghetti-like” structure. The vas can be absent at both sides, indicating a congenital abnormality [5,11]. Congenital bilateral absence of vas deferens (CBAVD) is associated with OA, and approximately 10% of these men have concurrent unilateral renal agenesis and should undergo an ultrasound scan to uncover this potentially health-threatening condition. By contrast, most patients (~60%) with congenital unilateral absence of vas deferens (CUAVD) are non-azoospermic [20]. A gene mutation associated with cystic fibrosis causes bilateral vas agenesis; therefore, genetic screening is advisable for the affected couples planning assisted reproductive technology (ART) [11,12]. Mutations affecting the cystic fibrosis transmembrane conductance regulator (CFTR) gene have also been identified in about 10% of men with CUAVD and normal kidneys, and it has been suggested that these patients should undergo CFTR testing as recommended for CBAVD patients. 

Assessment of the spermatic cord is mandatory as a varicocele may be found [5,11,21]. Varicocele is a prevalent congenital abnormality linked to infertility, impaired testicular growth, and hypogonadism [22,23,24,25]. Although varicocele is not uncommon in azoospermic men [24], it is debatable whether the vein dilation is coincident or contributory to spermatogenesis disruption in such patients [25]. Nonetheless, spermatogonia, spermatocytes, and spermatids are highly exposed to heat stress caused by varicocele. Furthermore, it was shown that varicocelectomy might ameliorate spermatogenesis and androgen production in azoospermic patients with spermatogenic failure [24,25,26].

The varicocele diagnosis is primarily made by a physical examination in a warm room with the patient standing. Palpable varicoceles are graded as (i) small (Grade 1): palpable during Valsalva maneuver, (ii) moderate size (Grade 2): palpable at rest, and (iii) large (Grade 3): visible and palpable at rest [22]. Scrotal Doppler ultrasound is indicated if a physical examination is inconclusive [11]. A maximum venous diameter of >3 mm in the upright position and during the Valsalva maneuver and venous reflux with a duration> 2 s usually correlate with the presence of a palpable varicocele [27].

### 2.3. Semen Analysis

The term azoospermia essentially refers to a semen analysis result. The assessment of an azoospermic ejaculate with normal volume (i.e., >1.5 mL) should be followed by examining the pelleted semen after centrifugation to rule out cryptozoospermia, defined by the presence of rare sperm [5,28]. Centrifugation should be carried out at 3000× *g* for 15 min or longer [29]. The finding of live sperm may allow ICSI to be carried out with ejaculated sperm, obviating surgical sperm harvesting. Azoospermia must be confirmed in at least two consecutive semen analyses because temporary azoospermia due to toxic, environmental, infectious, fever, or iatrogenic conditions can take place [30,31]. Assessment of azoospermic ejaculates on more than one occasion is also essential given the biological variability of the same individuals’ specimens. However, a limit of semen analyses (e.g., 2–3) might be set from a practical standpoint, although the exact number is difficult to ascertain. An interval between analyses is also advisable (e.g., one month apart) [32], albeit the optimal interval between examinations has not been established.

The state-of-art on how human semen should be assessed in the laboratory is set out by the World Health Organization (WHO), which periodically issues manuals that include standard operating procedures and reference values [29,31]. The lower reference limits (5th centile) for semen characteristics according to the 2010 WHO manual are as follows: (i) Semen volume: 1.5 mL, (ii) Total sperm number: 39 million/mL, (iii) Sperm concentration: 15 million/mL, (iv) Total motility: 40%, (v) Progressive motility: 32%, (vi) Vitality: 58% alive, and (vii) Sperm morphology: 4% normal forms [29].

Ejaculates of men with NOA-STF usually exhibit normal volume and pH (>7.2), indicating functional seminal vesicles and patent ejaculatory ducts [5]. By contrast, hypospermia (ejaculate volume < 1.5 mL) is typical in patients with HH-NOA [5,6]. A combination of a low volume (<1.5 mL), acidic ejaculate (pH < 7.2), with low fructose (e.g., <13 μmol per ejaculate) indicates seminal vesicle hypoplasia or obstruction [11]. Both conditions are associated with OA; the former with CBAVD and the latter with ejaculatory duct obstruction [33,34]. Seminal neutral alpha-glucosidase levels can also be determined as they reflect the epididymal function [29]. It was reported that seminal α-glucosidase levels < 18 mU/ejaculate is a reliable indicator of congenital bilateral absence of the vas deferens [4].

### 2.4. Hormonal Evaluation

Assessment of reproductive hormones’ serum levels may add relevant information to establish azoospermia type. Follicle-stimulating hormone (FSH) and testosterone are the essential hormones driving spermatogenesis [5,11]. Testosterone is produced by the Leydig cells under luteinizing hormone (LH) stimulation. Adequate levels of intratesticular testosterone are critical for sperm maturation [35]. By contrast, FSH is mainly responsible for increasing sperm production, and it collaborates with intratesticular testosterone to promote cell proliferation [36]. 

In general, there is an inverse relationship between FSH levels and spermatogonia quantity [37,38]. When spermatogonia number is absent or remarkably reduced, FSH levels increase; when spermatogonia number is normal, FSH levels are within normal ranges. FSH levels also relate to the proportion of seminiferous tubules exhibiting Sertoli cell-only on testicular biopsies [39]. Nevertheless, for patients subjected to sperm retrieval, FSH levels do not precisely predict whether spermatogenesis is present [40]. It is, therefore, possible to find focal sperm-producing areas in the testes of men with NOA-STF and elevated FSH levels during testicular sperm extraction [5,40,41,42,43]. 

Low FSH levels (e.g., <1.5 mIU/mL), combined with low LH (e.g., <1.5 mIU/mL), and low testosterone levels (e.g., <300 ng/dL) indicate primary or secondary HH [5,11]. In such cases, azoospermia is the result of an absence of testicular stimulation by pituitary gonadotropins. Pharmacotherapy using exogenous gonadotropins is highly effective in inducing sperm production in patients with congenital or acquired HH forms, with reported pregnancy rates of up to 65%, which are achieved naturally or with medically assisted reproduction [6,44].

Typically, patients with NOA-STF present with elevated FSH (>7.6 mIU/mL) and low testosterone (<300 ng/dL) levels, whereas those with OA show normal FSH and testosterone levels. Other hormones can also be assessed, including inhibin B, prolactin, estradiol, 17-hydroxyprogesterone, and sex hormone-binding globulin (SHBG) [11]. In particular, prolactin levels should be measured in patients with HH because prolactinoma may be the causative factor [11]. Inhibin-B levels reflect Sertoli cell integrity and spermatogenesis status [45]. However, its diagnostic value seems to be no better than that of FSH, and its use in clinical practice for azoospermia differentiation or sperm retrieval success prediction has not been broadly advocated [30,40].

### 2.5. Genetic Analysis

Azoospermia may have a genetic origin. The frequency of numerical autosomal and sex chromosome abnormalities, single-gene mutations, and partial or complete microdeletions of the Y-chromosome is increased in azoospermic patients [12,46]. Indeed, the incidence of genetic abnormalities increases as the sperm output decreases [47,48]. For instance, approximately 15% of men with NOA present with chromosomal anomalies, in contrast to ~5% of those with sperm concentration between 1 and 10 million/mL and <1% of men with >19 million/mL [49]. 

As a general rule, azoospermic men should undergo karyotype and Y chromosome microdeletion studies [5]. Exceptions apply to conditions in which azoospermia has an evident obstructive origin (e.g., vasectomy, ejaculatory duct obstruction) or a non-genetic-related etiology (e.g., post-chemotherapy/radiotherapy, post-orchitis). Karyotype and Y chromosome microdeletion tests are broadly available and are based on the screening of genomic deoxyribonucleic acid (DNA) taken mainly from peripheral blood samples. 

The most common abnormal karyotypic finding in azoospermic men is Klinefelter syndrome (KS), detected in ~10% of cases [12]. Azoospermia in KS men is associated with reduced testicular growth, pre-pubertal degeneration of primordial germ cells, or spermatogenic maturation arrest. For this reason, all azoospermic KS men have NOA-STF. Two karyotypic patterns are typically noticed: non-mosaic (47,XXY; ~85% of cases) and mosaic (47,XXY/46,X; ~15% of cases) [12]. Residual foci of active spermatogenesis is found on microdissection testicular sperm extraction (micro-TESE) in about 30–50% of KS men [12,40,50]. The retrieved sperm may be used for ICSI and generate a healthy child [28,40]. However, KS patients seem to be at an increased risk of having aneuploid gametes, which might increase the chance of producing offspring with a chromosomal abnormality [48]. Although the finding of an extra X chromosome is confirmatory, KS is suspected during the initial workup stages. These patients classically present with extremely small (1–8 mL) testes, gynecomastia (~40% of cases), and hypogonadism (e.g., scanty facial and pubic hair, poor libido, and erectile dysfunction) [5,11,12]. Reduced testosterone levels are commonly noticed (~80% of cases) and are attributed to decreased Leydig cell population due to the small testicular size. 

Microdeletions in the long arm of the Y chromosome are the second most common genetic cause of azoospermia [12]. This region aggregates 26 genes involved in spermatogenesis regulation, located in an interval named “AZF” (azoospermia factor); microdeletions at this interval are usually associated with azoospermia [38]. The AZF interval has three subregions, named AZFa, AZFb, and AZFc, each enclosing vital genes for spermatogenesis control. Approximately 10% of men with NOA-STF have microdeletions within the AZF interval that justify their condition [12,51]. 

The Y chromosome microdeletion study is based on a multiplex polymerase chain reaction (PCR), which amplifies Y chromosome sequences using specific sequence-tagged site primers [51]. Y-chromosome microdeletion testing allows detecting almost all clinically significant deletions. Hence, it helps identify the male infertility etiology, but it also provides information about treatment prognosis. Sperm retrieval success is determined by the type of Y microdeletion detected. Among men with AZFc deletions, sperm may be occasionally found in the ejaculate, or through testicular sperm extraction in at least 50% of individuals [5,51]. By contrast, patients with complete AZFa and/or AZFb microdeletions are not eligible for surgical sperm retrieval because large deletions involving these subregions are virtually incompatible with any residual spermatogenesis [5].

Notwithstanding the observations above, case reports showed sperm in the ejaculate of men with partial AZFb deletions [52,53]. While the AZFa region is relatively small and contains only two single-copy genes (*USP9Y* and *DDX3Y*), the AZFb and AZFc regions span over several megabase pairs and contain multiple relevant genes [51]. Notably, deletions usually remove more than one gene, but testing as currently performed only determines the presence or absence of a set of primers rather than gene-specific deletions. 

Azoospermic patients with AZFc microdeletions in whom testicular sperm are successfully retrieved can father a child through ICSI [5,40,54]. The probability of biological parenthood by ICSI appears to be not affected by the microdeletion. However, the male offspring of such fathers will inherit the genetic defect and, consequently, be infertile [54]. Genetic counseling is, therefore, recommended before sperm retrieval. Preimplantation genetic testing may be proposed for embryo sex selection to couples undergoing ICSI with testicular sperm retrieved from patients with AZFc microdeletions to avoid transmitting this form of infertility to the offspring. 

Cystic fibrosis transmembrane conductance regulator gene mutations usually result in CBAVD and, consequently, the affected patients have OA [12,55]. Over 2000 mutations have been discovered in the CFTR gene [56]. About eight out of ten patients with CBAVD harbor two CFTR mutations, usually in compound heterozygosity [57]. CFTR mutations were also implicated in bilateral epididymal obstruction in patients with palpable vasa. According to the 2020 European Association of Urology (EAU) guidelines on sexual and reproductive health, testing for CFTR gene mutations should be recommended for men with infertility and anatomical abnormalities of the vas deferens (unilateral or bilateral vas agenesis) when associated with normal kidneys [30]. In such cases, testing should be carried out in both partners of an infertile couple and has to include common point mutations (e.g., deltaF508, R117H, W1282X) and the 5T allele.

Screening for CFTR mutations is carried out in clinical molecular genetics laboratories. Methods for CFTR testing typically apply semiquantitative PCR analysis (e.g., multiplex ligation-dependent probe amplification) or quantitative fluorescent multiplex PCR [57]. The test report should be interpreted with prudence as not all mutations are implicated in disease. However, findings of mutations with clinical relevance confirm a genetic cause of OA [12]. In such patients, spermatogenesis is preserved, and therefore, sperm are easily retrieved from the testis or epididymis [8,33]. The retrieved sperm have to be used for ICSI, which results in adequate success rates [8,33]. The female partners should be screened for clinically relevant CFTR mutations. If the partner carries a CFTR mutation, the couple has up to a 50% risk of having a child with cystic fibrosis or CBAVD, depending on the parents’ type of mutation [11,30]. Preimplantation genetic testing may be offered for embryo sex selection or to identify non-affected embryos.

Given the solid genetic background of NOA, additional genetic analysis beyond karyotyping and screening for Y-chromosome microdeletions has been investigated. Gene panels using whole-exome sequencing have been proposed as a way to detect genetic variants possibly explaining NOA [56,58]. At present, however, these advanced genetic assessments are not entirely validated and therefore not yet suitable for inclusion in the routine investigation.

### 2.6. Imaging Studies

Imaging studies may add information to help determine the type and cause of azoospermia. 

Scrotal ultrasound (US) is useful to detect signs of testicular dysgenesis (e.g., microlithiasis, heterogeneous testis architecture) which are often related to NOA-STF [5]. As a general rule, men with suspected NOA-STF should undergo scrotal ultrasonography because these patients have an increased chance of testicular cancer [30]. A scrotal scan may also help to determine testis volume, epididymis characteristics, and presence of a varicocele if a physical examination is inconclusive (e.g., large hydrocele, inguinal testis, obesity) [11,21]. Additionally, indirect signs of obstruction might be seen during a scrotal US examination, including a dilated rete testis, enlarged epididymis, or absent/partially absent epididymis in patients with CBAVD [59]. Scrotal color Doppler US findings obtained from healthy fertile men provide reference ranges for clinicians [59,60]. For example, the lowest reference limit for testes volume (measured according to the ellipsoid formula) was about 12 mL, and thresholds for epididymis heal, tail, and vas deferens were 12, 6, and 4.5 mm [59].

Transrectal ultrasound (TRUS) is indicated in azoospermic patients with hypospermia (ejaculate volume < 1.5 mL) and seminal acidic pH if an obstruction is suspected [34]. Using TRUS, seminal vesicle abnormalities and prostatic cysts may be detected [34,59]. These lesions can obstruct the ejaculatory ducts and result in azoospermia [61]. Moreover, the presence of seminal vesical cysts should alert the clinician for possible concomitant genitourinary anomalies, including renal agenesis, dysgenesis, and autosomal dominant polycystic kidney disease [62,63]. Treatment to relieve the obstruction can be offered for these patients [8]. Besides, TRUS can help confirm CBAVD as the seminal vesicles of these patients are either absent or hypoplasic [11,28]. 

Magnetic resonance may also be used, and it is helpful to assess the distal parts of the seminal tract, the presence of prolactinomas, and an intra-abdominal location of an undescended testis [11]. Lastly, renal imaging studies should be performed in men with anatomical vas deferens abnormalities and no evidence of CFTR mutations. The unilateral absence of the vas deferens is usually associated with an ipsilateral absence of the kidney. Moreover, renal abnormalities (e.g., pelvic kidney) may be found in patients with bilateral absence of vas deferens without CFTR mutations [64]. 

## 3. Differential Diagnosis in Cases of Doubt: Testis Biopsy

Testis biopsy findings ultimately determine the type of azoospermia. However, from a practical standpoint, the differentiation is made in over 90% of cases using a detailed medical history, physical examination, semen analysis, hormonal assessment, and genetic and imaging studies [5,11,16]. Nevertheless, there are cases of doubt in which the differential diagnosis between OA and NOA remains undetermined unless a testis biopsy followed by histopathological analysis is carried out. 

Congenital intratesticular obstruction and congenital epididymal obstruction—unrelated to anatomic vas deferens abnormalities—cause OA, and these conditions are not easily recognizable [65]. Equally challenging to recognize is the functional obstruction of the distal parts of ejaculatory ducts [34,66]. Additionally, patients with idiopathic NOA might have normal FSH levels and normal testicular size (e.g., maturation arrest) because FSH levels correlate primarily with the number of spermatogonia [18,37]. A prediction model for testis histology in men with NOA showed that FSH levels could not correctly identify patients with maturation arrest [67].

A diagnostic testicular biopsy is the gold-standard method to discriminate OA from NOA in men with normal FSH, normal testicular size, and no apparent obstruction signs found in history, physical examination, semen analysis, and imaging studies. The biopsy should be ideally made using an open approach [30]. However, our experience with percutaneous biopsies—using a large needle (18 G) and a Cameco syringe holder—has been reassuring in the clinical scenario described above, as confirmed by the adequate amount of tissue extracted and the number of seminiferous tubules’ cross-sections examined. The extracted specimen is placed in a fixative solution, like Bouin’s, Zenker’s, or glutaraldehyde. Notably, formalin should not be used as a fixative because it disrupts tissue architecture. 

Histopathology results will inform if spermatogenesis impairment exists. Histopathology findings include (i) absent germ cells in seminiferous tubules (Sertoli cell-only), (ii) spermatogenic maturation arrest (incomplete spermatogenesis), (iii) presence of all spermatogenic stages, including spermatozoa, but with an evident impairment in germ cell number (hypospermatogenesis), (iv) tubular hyalinization, and (v) normal spermatogenesis [68,69]. Sertoli cell-only, maturation arrest, hypospermatogenesis, and tubular hyalinization are indicative of NOA. These patterns come alone or in combination (mixed pattern). By contrast, normal spermatogenesis is indicative of OA. 

Furthermore, intratubular germ cell neoplasia in situ (GCNIS) might be revealed in biopsy specimens taken from men with NOA-STF, mainly those with a history of cryptorchidism and/or multiple foci of testicular microlithiasis [30,70,71]. In general, GCNIS precedes the development of seminomas and non-seminoma tumors, and the risk of testicular cancer is increased in men with NOA [72].

Notably, diagnostic biopsies might harm the testis; therefore, they should be limited to very selected cases. Its routine use as a diagnostic tool to establish the azoospermia type is not recommended by relevant guidelines [30,32]. In our settings, one or more specimens are extracted and examined fresh in the in vitro fertilization (IVF) laboratory during a diagnostic biopsy [68,69,73]. In the presence of viable sperm, cryopreservation is offered [73,74,75,76]. Our approach is consistent with the EAU guidelines recommendations [30], stating that a biopsy should be combined with testicular sperm extraction (TESE) for possible sperm cryopreservation. Cryopreservation is carried out using isolated sperm suspensions or tissue fragments [74,75,77]. 

Along these lines, a formal scrotal exploration might be applied to identify an obstruction at the epididymis or proximal vas deferens level that could be ultimately treatable using microsurgery (e.g., vasoepididymostomy) at the same operative time [9]. In the above scenario, a testis biopsy should be taken and examined fresh to confirm the presence of active spermatogenesis. Moreover, even if signs of obstruction are evident and a reconstructive procedure is carried out, a testis specimen should be sent for formal histopathology examination as good clinical practice. 

In cases of untreatable epididymal obstructions, microsurgical epididymal sperm aspiration may be applied to harvest sperm for cryopreservation [7,9,10,78,79]. By contrast, a testicular sperm retrieval technique (e.g., conventional TESE or microdissection TESE) should be carried out in the same operative time if no signs of obstruction are seen [10]. During the sperm extraction, a specimen should also be taken for histopathology examination to confirm the type of azoospermia. 

**Figure 1:**
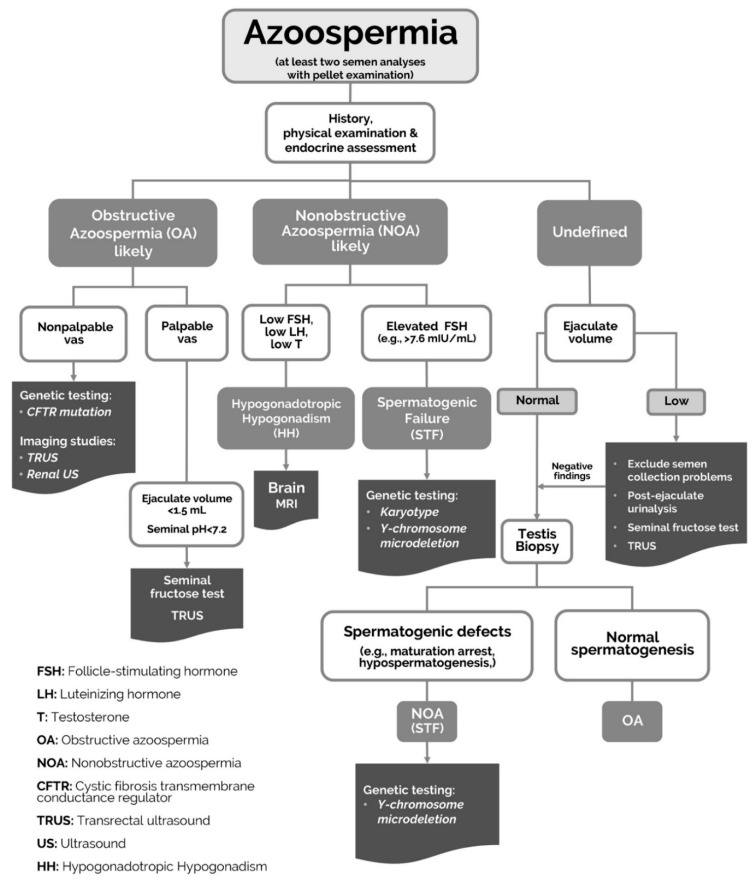
Algorithm for azoospermia differential diagnosis.

A clinical algorithm to help distinguish OA from NOA related to HH or STF is provided in Figure 1.

## 4. Clinical Cases: Difficult Differential Diagnosis 

### 4.1. Case 1

A 36-year-old man presented for evaluation with a 7-year infertility history and azoospermia confirmed on multiple semen analyses. His wife was 27 years old and had no obvious female factor (e.g., eumenorrheic, patent tubes, normal-sized ovaries, normal ovarian reserve (Anti-Müllerian Hormone level of 2.5 ng/mL), no previous surgery, no medical comorbidities). 

His childhood and adolescent history were unremarkable. In the sexual history, the patient complained of decreased libido and mild erectile dysfunction, which resulted in an irregular intercourse routine. He denied previous or current gonadotoxic exposure, medication use, or sexually transmitted diseases. However, he reported a history of a right-sided hernia repair at age 26 and noticed that the size of the right testis decreased after the operation. 

Physical examination revealed a normal virilized man with no gynecomastia, a body mass index (BMI) of 30.1 kg/m^2^, and a right inguinal scar from previous hernia repair. His right testis was atrophic (Prader orchidometry of 2 mL), whereas his left testis had a normal size (Prader orchidometry between 15 and 20 mL). The right epididymis was reduced in size, and the left epididymis was normal on palpation. Both vas deferens were palpable, and we did not detect varicocele on physical examination. Fasting blood tests taken in the morning (~10:00 a.m.) revealed a serum FSH level of 6.1 mIU/mL (reference: 1.4–8.1), LH level of 5.6 mIU/mL (reference: 1.5–9.3), estradiol level of 30.3 pg/mL (reference: <39.8 pg/mL), thyroid-stimulating hormone level (TSH) of 2.6 µIU/mL (reference range: 0.48–5.60 µIU/mL), thyroxin (T4) level of 0.99 ng/dL (reference: 0.85–1.50 ng/dL), prolactin level of 7.1 ng/mL (reference range: 2.1–17.7 ng/mL), total testosterone level of 266 ng/dL (reference range: 241–827 ng/dL), free testosterone level of 5 ng/dL (reference range: 3.03–14.8 ng/dL), and vitamin D of 52 ng/mL (reference: >20 ng/mL). Two additional semen analyses performed in the fertility clinic’s andrology laboratory confirmed the presence of azoospermia after the examination of the centrifuged pellet, and these ejaculates had normal volume (4 and 3 mL) and pH (8.0 and 7.8). Genetic tests were ordered, which reported a normal (46,XY) karyotype and no Yq chromosome microdeletions. 

**Figure 2:**
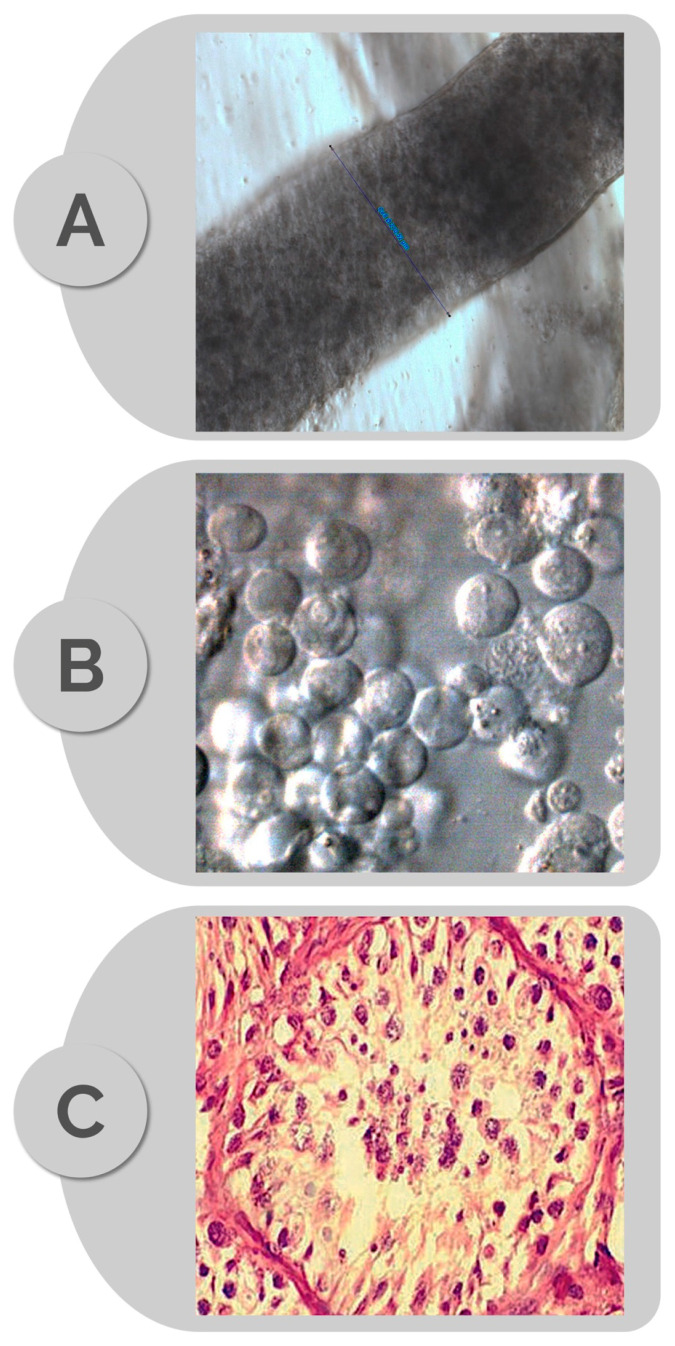
Photomicrographs illustrating: (**A**) intact seminiferous tubule (diameter 270 micrometers), (**B**) cell suspension obtained after mechanical tubule mincing, and (**C**) corresponding histopathology (hematoxylin/eosin) specimen revealing germ cell maturation arrest (MA). Images A and B obtained at 400× magnification using an inverted optical microscope (Nikon Eclipse Diaphot 300, Nikon, Japan, with phase contrast (Hoffman)).

Although the diagnosis of right testicular atrophy secondary to iatrogenic vascular damage during hernia repair was established, the type of azoospermia on the left testis was more equivocal. Therefore, a percutaneous testicular biopsy was undertaken on the left testis at the fertility center’s operating theater and sent for both fresh and histopathology examinations (Figure 2). The fresh specimen contained abundant germ cells but no mature sperm or elongated spermatid (Figure 2B). The histopathology specimen revealed maturation arrest at the spermatocyte stage in all tubules examined (120 cross-sections) (Figure 2C).

With the diagnosis of NOA due to maturation arrest on the left testis, we recommended sperm retrieval. However, the patient was advised to undergo an off-label hormonal modulation, which seems justified in selected NOA cases, particularly those associated with hypogonadism [5,80,81]. He was started on human chorionic gonadotropin (recombinant hCG, 125 mcg twice weekly). After two months of treatment, his total testosterone levels improved to 476 ng/dL, his FSH levels dropped to <1.5 mIU/L, and his estradiol levels raised to 55 pg/mL. He was then started on FSH (recombinant FSH 150 IU twice weekly) and anastrozole 1 mg/day. Therapy lasted for six months, and during treatment, no sperm were found on the follow-up semen analysis. 

He was then subjected to micro-TESE on the left side. At the time of surgery, his hormone levels were: FSH of 3.2 mIU/L, total testosterone of 578 ng/dL, and estradiol of 39 pg/mL. During the operation, we were able to harvest viable sperm with apparent adequate morphology from the seminiferous tubules, which were cryopreserved using conventional and vitrification methods [73,74]. A specimen taken for histopathology showed germ cell maturation arrest with focal areas of normal spermatogenesis. Subsequently, sperm injections were performed with frozen-thawed testicular sperm. At oocyte pick-up, seven metaphase-II oocytes were retrieved, five of which fertilized, and three developed until the blastocyst stage. A single embryo transfer was performed, which resulted in a term delivery of a baby boy at term. Two blastocysts remain cryopreserved.

### 4.2. Case 2

A 35-year-old man presented for evaluation with an 8-year infertility history and azoospermia confirmed on multiple semen analyses. His partner was 32 years old, eumenorrheic, with no evident female factor or medical co-morbidities, despite an ovarian reserve in the lower normal limits (Anti-Müllerian Hormone level of 1.2 ng/mL). 

The couple’s sexual history was unremarkable, as was the patient’s childhood and adolescent medical history. He denied previous or current gonadotoxic exposure, medication use, or sexually transmitted diseases. The patient had a history of bilateral varicocele repair at age 27, with no apparent complications. 

Physical examination revealed a normal virilized man with no gynecomastia, a BMI of 32.5 kg/m^2^, and a bilateral inguinal scar from the previous varicocelectomy. His testes were found to have normal volume (Prader orchidometry of 15 cc). The epididymides were normal, and the vas deferens was palpable on both sides. Fasting blood tests taken in the morning (~10:00 a.m.) revealed a serum FSH level of 4.4 mIU/mL (reference range: 1.4–8.1), LH level of 3.8 mIU/mL (nl: 1.5–9.3), estradiol level of 28 pg/mL (reference: <39.8 pg/mL), TSH of 1.2 µIU/mL (reference range: 0.48–5.60 µIU/mL), T4 level of 1.1 ng/dL (reference: 0.85–1.50 ng/dL), prolactin level of 5.8 ng/mL (reference range: 2.1–17.7 ng/mL), total testosterone level of 360 ng/dL (reference range: 241–827 ng/dL), and free testosterone level of 8.8 ng/dL (reference range: 3.03–14.8 ng/dL). 

Two semen analyses carried out in the fertility center’s andrology laboratory confirmed the presence of azoospermia after the examination of the centrifuged specimens, and these ejaculates had normal volume (>1.5 mL) and pH (>7.2). The genetic analysis revealed a normal (46,XY) karyotype and absence of Yq chromosome microdeletions. 

The differential diagnosis remained equivocal, and therefore the patient had a scrotal exploration, which revealed no signs of obstruction. A right micro-TESE was carried out in the same operative procedure. The examination of the seminiferous tubules showed a homogeneous pattern of healthy tubules. Random micro-biopsies were taken for fresh examination, which revealed abundant germ cells, typical of maturation arrest, and no mature sperm or elongated spermatid. We decided to terminate the operation without exploring the contralateral testis. A specimen was taken and sent for histopathology, which confirmed maturation arrest at the primary spermatocyte stage.

Four weeks postoperatively, the patient was started on human chorionic gonadotropin (recombinant hCG 125 mcg twice weekly) and FSH (recombinant FSH 150 IU twice weekly). His hormone levels were monitored monthly and medication adjusted whenever needed, with the goal to keep testosterone levels between 500 and 800 ng/dL and FSH levels within normal levels. Semen analyses were also performed from the third month of therapy onwards, and after five months of therapy, occasional motile sperm were found, all of which were morphologically abnormal (mainly globozoospermic sperm). Sperm cryopreservation was carried on several occasions, and the couple had an ICSI cycle performed with frozen-thawed ejaculated testicular sperm. Sperm injections were carried out in 7 metaphase II oocytes, two of which fertilized, and one day-3 embryo was replaced into the uterus, but implantation did not occur. The embryologists informed that the quality of sperm was unsuitable for ICSI. 

We opted to continue with medication and proceed to micro-TESE on the left testis, which was carried out after 12 months of gonadotropin therapy. At the time of micro-TESE, his hormone levels were: FSH of 5.6 mIU/L, total testosterone of 738 ng/dL, and estradiol of 46 pg/mL. Mature sperm were found intraoperatively; however, all harvested sperm exhibited abnormal morphology, as seen in the cryptozoospermic semen analyses. ICSI was performed in five metaphase-II oocytes, two of which fertilized with fresh testicular sperm isolated from the micro-TESE procedure. These zygotes developed into embryos, which were replaced in the partner’s uterus on the third day of development. Again, no pregnancy was obtained. The couple declined the offer to carry on with donor sperm insemination. To our knowledge, at the time of writing, the couple remained childless.

### 4.3. Case 3 

A 40-year-old man presented to the fertility clinic with a 4-year infertility history and azoospermia confirmed on repeated semen analyses. His wife was 29 years old and had adequate ovarian reserve markers, patent tubes, and normal gynecological investigations. 

The couple’s sexual history was mostly unremarkable, although the patient complained of occasional perineal discomfort after ejaculation. His childhood and adolescent medical history were also not significant. He had previous chickenpox at age 12 but denied a history suggestive of mumps orchitis. The patient underwent typical pubertal changes and denied sexually transmitted diseases, previous/current medication use, or gonadotoxic exposure, except cigarette smoking since age 19. He also denied previous surgeries. The only possible relevant finding was his habit of equestrian sports, which he practiced at least twice a week since age 16. 

Physical examination revealed a normal virilized man with no gynecomastia, BMI of 29.4 kg/m^2^, no inguinal /scrotal scars, and normal-sized testicles (Prader orchidometry of 20 cc). The epididymides had normal characteristics, the vasa deferentia were palpable, and the spermatic cords had no signs of varicocele; however, a small hydrocele was noted on both hemiscrotum. Fasting blood tests taken in the morning revealed a serum FSH level of 6.2 mIU/mL (reference range: 1.4–8.1), LH level of 3.6 mIU/mL (nl: 1.5–9.3), estradiol level of 23 pg/mL (reference: <39.8 pg/mL), TSH level of 2.1 µIU/mL (reference range: 0.48–5.60 µIU/mL), T4 level of 1.2 ng/dL (reference: 0.85–1.50 ng/dL), prolactin level of 13.5 ng/mL (reference range: 2.1–17.7 ng/mL), total testosterone level of 418 ng/dL (reference range: 241–827 ng/dL), and free testosterone level of 11.5 ng/dL (reference range: 3.03–14.8 ng/dL). 

Semen analysis was carried out in the fertility center’s andrology laboratory, which confirmed azoospermia after examining the centrifuged specimen. The ejaculate had a normal pH (8.0), but its volume was at the lower normal limits (1.5 mL). A TRUS was ordered to evaluate the complaint of perineal discomfort and borderline semen volume further, but its results were not suggestive of any signs of obstruction. A post-ejaculation urinalysis was also performed to check for retrograde ejaculation, yet no sperm were found. Additionally, a scrotum ultrasound confirmed the physical exam findings, but it did not add any relevant information to ascertain whether the azoospermia was obstructive or nonobstructive. The genetic analysis revealed a normal (46,XY) karyotype and no Yq chromosome microdeletions. 

The patient had a scrotal exploration that revealed bilateral epididymal obstruction signs, possibly idiopathic or post-traumatic (equestrian sports). The testicles and the vasa deferentia were normal, and small-volume hydroceles were indeed present. A healthy epididymal tubule was isolated and incised using a microsurgical technique. Subsequently, the fluid was collected and sent to the laboratory for examination, revealing abundant motile sperm. The harvested epididymal sperm were cryopreserved. A microsurgical vasoepididymostomy was carried out using the intussusception technique applying three double-arm sutures in a triangular fashion [9]. The procedure was repeated on the contralateral side. Testicular specimens taken for histopathology showed normal spermatogenesis. 

Follow-up semen analyses at 6 and 9 months postoperatively revealed a sperm concentration of 7 and 12 million/mL, 29% and 38% progressive motility, 4% and 7% strict morphology, and 4.7 and 9.2 million total motile sperm count, respectively. The couple achieved natural pregnancy one year postoperatively, which resulted in the delivery of a healthy baby girl at 38 gestational weeks.

## 5. Discussion 

The clinical assessment and management of infertile men with azoospermia should consider the (i) differential diagnosis of azoospermia, (ii) identification of patients eligible for reconstructive procedures (e.g., OA), gonadotropin therapy (e.g., NOA-HH), or sperm retrieval, (iii) identification of patients with NOA-STF that might benefit from interventions (e.g., varicocele repair, hormonal modulation) before a sperm retrieval attempt, (iv) use of an optimal surgical method to harvest sperm, and (v) utilization of state-of-the-art IVF techniques when applicable. A detailed discussion of these aspects is outside the scope of this article and can be found elsewhere [5,9,10].

Nevertheless, the most challenging azoospermic patient to manage clinically is probably that with NOA-STF. Table 2 outlines the essential aspects to be considered under this scenario. Among several critical factors, a sensitive matter relates to hormonal modulation for men with NOA, as briefly described in clinical cases 1 and 2. The reason stems from the fact that it is generally believed that empirical medical treatment for men with NOA-STF is ineffective because gonadotropins’ plasma levels are usually high. Yet, many patients with NOA-STF present with hypogonadism and might thus lack adequate levels of intratesticular testosterone, which are essential for spermatogenesis in combination with adequate Sertoli cell stimulation by FSH [80,81,82]. Furthermore, gonadotropin action is determined by the frequency, amplitude, and duration of its secretory pulses. Due to the high baseline levels of endogenous gonadotropins commonly seen in patient with NOA-STF, the relative amplitudes of FSH and LH are low, leading to a paradoxically weak stimulation of Leydig and Sertoli cells [35,83]. Therefore, there may be a potential role for pharmacotherapy in men with NOA [84,85]. 

Selective estrogen receptor modulators, aromatase inhibitors, human chorionic gonadotropin (hCG), and FSH have been used off-label to manipulate male reproductive hormones and optimize intratesticular testosterone production [5,84,86,87,88]. The goals are to induce recovery of sperm to the ejaculate or improve surgical sperm retrieval rates. Case series and a few cohort studies suggested that these treatments might increase sperm retrieval rates, and in some cases, treatment was associated with the return of minimal numbers of sperm to the ejaculate [5,84,85,86,87,88]. Despite that, no randomized controlled trial exists, making it difficult to make clear recommendations on this matter.

Notwithstanding these observations, limited data indicate that treatment with hCG and recombinant FSH could lead to 10–15% higher sperm retrieval rates than sperm retrieval with no previous treatment [35]. Furthermore, hCG treatment was shown to improve intratesticular testosterone production remarkably in men with NOA [89]. Based on these concepts and with the goals of inducing return of sperm to the ejaculate or improving surgical sperm retrieval rates, we have used hCG alone or in combination with recombinant FSH off-label to optimize intratesticular testosterone production and FSH action, as previously described [5]. Our treatment protocol, utilized in clinical cases 1 and 2, relies primarily on hCG to boost intratesticular production. Additionally, hCG treatment was shown to decrease FSH levels, which are typically elevated in most of these patients [51]. Based on limited data from animal and human studies, it has been speculated that FSH reset to normal levels might reduce Sertoli cell desensitization caused by excessive circulating endogenous gonadotropins [90,91,92,93,94,95]. Consequently, an increased Sertoli cell function and expression of FSH receptors could be obtained. Our patients are followed with a monthly hormonal assessment, and we add an aromatase inhibitor in the course of treatment when the testosterone (ng/dL) to estradiol (pg/mL) ratio becomes less than 10. We also prescribe recombinant FSH when, after hCG treatment, the FSH levels drop below 1.5 IU/L [4,51]. The ultimate goal is to increase intratesticular testosterone to optimal levels through hCG stimulation while securing adequate FSH levels within normal ranges. Although we need more data in this area, hormone stimulation for men with NOA may be worth considering in selected cases. 

## 6. Conclusions

The differential diagnosis between OA and the two forms of NOA, namely NOA-STF and NOA-HH, can be effectively established in most patients based on a standardized male infertility workup. This is the first and critical step in the clinical decision-making process, and it will guide the physician on how to optimally manage these patients, thus providing the couples with an optimal path for parenthood. 

A testicular biopsy should be reserved for the cases of doubt, mainly in patients whose history, physical examination, semen analysis, hormonal evaluation, genetic tests, and imaging studies are inconclusive. Histopathology findings will indicate if spermatogenesis is preserved or disrupted, confirming whether azoospermia is obstructive or nonobstructive. Besides providing specimens for a formal histopathology examination, a diagnostic testis biopsy allows for a concomitant fresh examination of one or more extracted specimens; in the presence of viable sperm, cryopreservation should be offered. Alternatively, a formal surgical scrotal exploration may be utilized in cases of doubt, provided the surgeon is prepared to fix an obstruction at the level of epididymis or vas deferens or perform epididymal or testicular sperm retrieval as appropriate. Therefore, these procedures should be carried out at properly equipped facilities.

A coordinated multidisciplinary effort involving reproductive urologists/andrologists, reproductive gynecologists, geneticists, and embryologists is vital to offer infertility patients with azoospermia the best chance of achieving biological parenthood.

## Figures and Tables

**Table 1:** Medical history outline. Adapted from Esteves et al. [11], Clinics 66, 691–700, 2011.

Elements	Components
(1) Infertility History	Age of partners, length of time the couple has been attempting to conceive Contraceptive methods/duration Previous pregnancy/miscarriage (current partner/partner/another partner) Previous treatments Treatments/evaluations of female partner
(2) Sexual History	Potency, libido, lubricant use Ejaculation, timed intercourse, frequency of masturbation
(3) Childhood and Development	Cryptorchidism, hernia, testicular trauma, testicular torsion, infection (e.g., mumps) Sexual development, puberty onset
(4) Personal History	Systemic diseases (e.g., diabetes, cirrhosis, hypertension) Sexually transmitted diseases, tuberculosis, viral infections, genital and systemic bacterial infections, history of fever
(5) Previous Surgery/Treatment	Orchidopexy, herniorrhaphy, orchiectomy (e.g., testicular cancer, torsion) Retroperitoneal and pelvic surgery Other inguinal, scrotal, or perineal surgery Bariatric surgery, bladder neck surgery, transurethral resection of the prostate
(6) Gonadotoxin Exposure	Pesticides, alcohol, cocaine, marijuana Medication (e.g., chemotherapy agents, cimetidine, sulfasalazine, nitrofurantoin, allopurinol, colchicine, thiazide, α- and β-blockers, calcium blockers, finasteride) Organic solvents, heavy metals Anabolic steroids, tobacco use High temperatures, electromagnetic energy Radiation (e.g., therapeutic, nuclear power plant workers)
(7) Family History	Cystic fibrosis, endocrine diseases Infertility
(8) Current Health Status/Lifestyle	Respiratory infection, anosmia Galactorrhea, visual disturbances Obesity, metabolic syndrome

**Table 2:** Interventions and recommended actions in the clinical management of azoospermic men with nonobstructive azoospermia seeking fertility.

Clinical Management Step	Intervention	Action	Interpretation
Differential diagnosis	Medical history, physical examination, endocrine profile (FSH and testosterone levels at a minimum; LH, prolactin, thyroid hormones, 17-hydroxiprogesterone and estradiol are added as needed), and examination of pelleted semen on multiple occasions. Testicular biopsy could be considered in selected cases in which the differential diagnosis could not be determined.	Confirm that azoospermia is due to spermatogenic failure, and identify men with severely impaired spermatogenesis having few sperm in the ejaculate (cryptozoospermia).	A differential diagnosis between obstructive azoospermia, hypogonadotropic hypogonadism, and spermatogenic failure should be established as management varies according to the type of azoospermia.
Determination of proper candidates for sperm retrieval	Y chromosome microdeletion screening using multiplex PCR blood test. The basic set of PCR primers recommended by the EAA/EMQN for the diagnosis of Yq microdeletion includes: sY14 (SRY), ZFX/ZFY, sY84 and sY86 (AZFa), sY127 and sY134 (AZFb), sY254, and sY255 (AZFc).	Deselect men with microdeletions involving subregions AZFa, AZFb, and AZFb+c.	Approximately 10% of men with NOA-STF harbor microdeletions within the AZF region. SR success in men with YCMD involving the subregions AZFa, AZFb, and AZFb+c are virtually nil, and such patients should be counseled accordingly. SR success in men with AZFc deletions range from 50% to 70%. Genetic counseling should be offered to men with AZFc deletions because testicular spermatozoa used for ICSI will invariably transmit the deletion from father to son.
Identification of patients who could benefit from medical therapy or varicocele repair before sperm retrieval	Serum levels of FSH, total testosterone and estradiol.	Consider medical treatment with gonadotropins, aromatase inhibitors, or selective estrogen receptor modulators for NOA-STF patients with hypogonadism (TT < 300 ng/dL) or T/E ratio < 10. FSH therapy might be needed if FSH drop to below 1.5 mIU/mL during hCG treatment.	Patients should be informed that the evidence of a positive effect of medical treatment remains equivocal.
	Physical examination to identify the presence of clinical varicocele and analysis of testicular biopsy results (if available)	Consider microsurgical repair of clinical varicocele.	Microsurgical varicocele repair is associated with better outcomes concerning recurrence and postoperative complications. Patients with testicular histopathology indicating Sertoli cell-only are unlikely to benefit from varicocele repair. Evidence of a positive effect of varicocele repair is limited, and patients should be counseled accordingly.
Selection of the most effective surgical method for testicular sperm acquisition	Analysis of testicular biopsy results (if available) and of whether sperm have been obtained in previous treatment and by which method.	Microdissection testicular sperm extraction. Conventional testicular sperm extraction may be considered in cases of previous success with TESE, particularly when testicular histopathology indicates hypospermatogenesis.	Micro-TESE in NOA-STF is associated with higher SR success than conventional TESE. The lower tissue removal facilitates sperm processing and lessens testicular damage.
State-of-the-art laboratory techniques to handle surgically extracted testicular spermatozoa	Extraction of a minimum volume of tissue by micro-TESE facilitates tissue processing and search for sperm. Testicular tissue preparation techniques include mechanical and enzymatic mincing and erythrocyte lysis.	Sterile techniques, stable pH and temperature, and high laboratory air quality conditions are helpful to optimize micromanipulation efficiency and safety assurance. Excess sperm not used for ICSI should be cryopreserved for future attempts.	Spermatozoa collected from NOA-STF men are often compromised in quality and are more fragile than ejaculated counterparts. The reproductive potential of such gametes used for ICSI is differentially affected by NOA-STF.

EAA: European Association of Andrology; EMQN: European Molecular Genetics Quality Network; AZF: azoospermia factor; FSH: Follicle-stimulating hormone; ICSI: intracytoplasmic sperm injection; LH: luteinizing hormone; micro-TESE: microdissection testicular sperm extraction; NOA-STF: nonobstructive azoospermia due to spermatogenic failure; PCR: polymerase chain reaction; SR: sperm retrieval; T/E: testosterone to estradiol ratio; TESE: testicular sperm extraction; TT: total testosterone; YCMD: Y-chromosome microdeletions. Adapted from Esteves [5], Asian J. Androl. 17, 459–470, 2015, an Open Access article distributed under the terms of the Creative Commons Attribution Non-Commercial License.

## Data Availability

All data related to this manuscript are provided in the text.
